# Impact of manual therapy in children with Adolescent Idiopathic Scoliosis

**DOI:** 10.1186/1748-7161-8-S2-P8

**Published:** 2013-09-18

**Authors:** Stefanie Reid, Channing Tassone, John Thometz, XueCheng Liu

**Affiliations:** 1 Aegis Therapies, Milwaukee, WI, USA

## Background

Using a manual therapy and a motor relearning approach to treatment of adolescent idiopathic scoliosis (AIS) operates from the premise that it is related to muscle imbalances at the core spinal stabilizers. The therapy will result in decreased lateral decompensation at the trunk and assist in improved postural alignment and stability of the spine. 

## Purpose

The goal of this study was (1) to determine the correction of spinal curvature, shoulder girdle, pelvic obliquity and 3-dimensional back contour deformities following the use of manual therapy and (2) to investigate changes of shoulder girdle, core and hip muscle strengths. 

## Methods

We recruited 11 children with AIS, with a mean age of 14 years of age. Children with AIS who received manual therapy and the motor relearning approach were followed for an average of 4.5 months. Physical therapy intervention consisted of 60-minute treatment sessions two times per week. An impairment-based manual physical therapy method was utilized, consisting of passive force application and specific mobilization and manipulation techniques along planes of movement parallel or perpendicular to the anatomic planes of joint surfaces. Our motor relearning approach consisted of a segmentation approach to postural control, performed during specific functional and exercise-based tasks. The 3-dimensional back contour was measured using the Milwaukee Topographic System (MTS) before and after the therapy. The Wilcoxon Sign Rank test was used for comparison of muscle strength and 3-dimensional back contour variables before and after the therapy. 

## Results

Except that the quadratus lumborum muscle did not show an increased strength, other muscles significantly improved strength, ranging from 3.7 to 4.5 (P<0.01). There were essentially no changes in the analog angle to Cobb in the coronal plane (from 8.4º to 8.3º). Differences were noted pre- and post-therapy with shoulder discrepancy, rotation in the transverse plane (axial surface rotation and Suzuki score), kyphosis and lordosis, the midline deviations and angles as well as the back height, but these changes never reached statistical significance (P>0.05). 

## Conclusions and discussion

A short-term follow-up of the manual therapy and motor relearning approach significantly increased strength at abdominals, shoulder girdle and hip. Asymmetries in shoulder girdle and rib humps will be further investigated. 

